# Does Central Statistical Monitoring Improve Data Quality? An Analysis of 1,111 Sites in 159 Clinical Trials

**DOI:** 10.1007/s43441-024-00613-w

**Published:** 2024-02-09

**Authors:** Sylviane de Viron, Laura Trotta, William Steijn, Steve Young, Marc Buyse

**Affiliations:** 1 CluePoints S.A, Avenue Albert Einstein, 2a 1348 Louvain-la-Neuve, Belgium; 2 CluePoints Inc, King of Prussia, USA; 3 International Drug Development Institute (IDDI), Louvain-la-Neuve, Belgium; 4 Interuniversity Institute for Biostatistics and Statistical Bioinformatics (I-BioStat), Hasselt University, Hasselt, Belgium

**Keywords:** Statistical monitoring, Central monitoring, Risk-based quality management, Risk-based monitoring, RBM, RBQM, Clinical trial quality, Data quality assessment, Site performance

## Abstract

**Background:**

Central monitoring aims at improving the quality of clinical research by pro-actively identifying risks and remediating emerging issues in the conduct of a clinical trial that may have an adverse impact on patient safety and/or the reliability of trial results. This paper, focusing on statistical data monitoring (SDM), is the second of a series that attempts to quantify the impact of central monitoring in clinical trials.

**Material and Methods:**

Quality improvement was assessed in studies using SDM from a single large central monitoring platform. The analysis focused on a total of 1111 sites that were identified as at-risk by the SDM tests and for which the study teams conducted a follow-up investigation. These sites were taken from 159 studies conducted by 23 different clinical development organizations (including both sponsor companies and contract research organizations). Two quality improvement metrics were assessed for each selected site, one based on a site data inconsistency score (DIS, overall -log_10_ *P*-value of the site compared with all other sites) and the other based on the observed metric value associated with each risk signal.

**Results:**

The SDM quality metrics showed improvement in 83% (95% CI, 80–85%) of the sites across therapeutic areas and study phases (primarily phases 2 and 3). In contrast, only 56% (95% CI, 41–70%) of sites showed improvement in 2 historical studies that did not use SDM during study conduct.

**Conclusion:**

The results of this analysis provide clear quantitative evidence supporting the hypothesis that the use of SDM in central monitoring is leading to improved quality in clinical trial conduct and associated data across participating sites.

## Introduction

For years, regulatory agencies such as FDA and EMA have required that the conduct and the progress of clinical trials be monitored to ensure patient protection and the reliability of trial results [1, 2]. Until recently, the primary approach to meeting this requirement included frequent visits to each investigative site by designated site monitors who manually reviewed all of the patient source data to ensure it was reliably reported to the trial sponsor—a practice known as 100% source data verification (SDV) [3–7]. However, a major revision to the ICH GCP guidance was published in 2016 which encouraged the use of central monitoring to support a more effective and efficient approach to monitoring trial conduct across all sites [8].

Central monitoring, which is a component of risk-based quality management (RBQM), aims to detect emerging quality-related risks (either pre-identified or unanticipated risks) proactively during a clinical trial, resulting in study team mitigating risks and addressing any confirmed issues and therefore drive higher-quality outcomes [1]. By doing so, central monitoring limits the amount of data impacted by confirmed issues and prevents these issues from affecting future data.

The term “quality” as used here and throughout this paper is intended to refer to the extent to which the observed conduct of a study is contributing to the GCP imperative of “human subject protection and reliability of trial results.” [8].

A variety of tools may be applied to support central monitoring, but the following two methods are most commonly used [9]:

- Statistical Data Monitoring (SDM)—The execution of a number of statistical tests against some or all of the patient data in a study, which are designed to identify highly atypical data patterns at sites that may represent various systemic issues in the conduct of the study. The types of issues identified may include fraud, inaccurate data recording, training issues, and study equipment malfunction or miscalibration [1, 3, 10–15].
- Key Risk Indicators (KRIs)—Metrics that serve as indicators of risk in specific targeted areas of study conduct. Sites that deviate from an expected range of values (i.e., risk thresholds) for a given KRI are flagged as “at risk.” The risk thresholds can be discrete values (e.g., procedure compliance rate < 10%) or statistically determined (e.g., *P*-value < 0.05) based on a comparison of data between the site and the trend across all sites in the study [1, 10–13, 16]. Note that quality tolerance limits (QTLs) as referenced in ICH E6 (R2) are quite similar in concept to KRIs and may be considered a designated type of study-level KRI [17].

Few analyses to date have assessed the impact and the performance of central monitoring for a large pool of studies. There are examples of analyses focusing on the quantitative performance of central monitoring but they have typically analyzed one or only a few datasets or studies retrospectively and generally used simulated data [6, 18, 19]. Papers analyzing ongoing studies have more often explored the adoption of central monitoring rather than its impact on quality [9]. Hence, efforts are still required to quantify the impact of central monitoring on improving quality in clinical trials.

This paper is the second of a series that aims to quantify the impact of central monitoring on quality in clinical studies. The first paper focused on KRIs and found that the use of KRIs is leading to quality improvement in the majority of at-risk study sites (83%) [16]. This paper presents the results of an analysis of quality improvement metrics associated with the use of SDM as part of central monitoring. Specifically, our hypothesis is that the detection of risks (i.e., potential issues) identified through the use of SDM and acted upon by study teams result in higher levels of quality as measured by two metrics that are described in the ‘Quality Improvement Analysis’ section of this article. To assess whether the obtained results might be due to random play of chance, we further applied the same methods to two studies available to us that did not use central monitoring—neither SDM nor KRIs—during the conduct phase of the studies. While the number of studies available for comparison was small, it provided an interesting set of data and results for comparison with the primary analysis.

## Materials and Methods

### Central Monitoring Solution

A central monitoring software built on statistical algorithms [3–7] was used to generate the data for this analysis. The platform was launched in 2015 to support various RBQM processes, including central monitoring and SDM [14, 16].

Central monitoring including SDM typically involves the analysis of data at regular intervals (e.g., monthly) during the conduct of a study. SDM analyzes clinical data collected from various sources, including electronic Case Report Forms (eCRFs), central laboratories, electronic Patient-Reported Outcome (ePRO) and electronic Clinical Outcome Assessment (eCOA) systems, and wearable technologies. When SDM identifies a site that has exceeded a risk alert threshold (e.g., *P*-value < 0.05) for a specific statistical test, the system triggers the creation of a risk signal for review and follow-up by members of the study team. Based on an initial review of the risk signal, the study team decides to close it directly if they conclude that it does not represent an actual issue or to open it if further investigation and/or remediation is needed. A risk signal typically remains open until the study team determines that it is either resolved or no longer applicable (e.g., site or study closure and inability to remediate) [16]. All risk signals are closed by the end of the study.

The SDM tool referenced in this analysis applies a battery of standard statistical tests that detect sites with atypical data patterns compared to all study sites [3–7]. The approach is considered “unsupervised” since the same set of standard tests run against all of the clinical data for each study and not pre-directed by a study-specific assessment of risk. Additionally, the tool computes a “Data Inconsistency Score” (DIS) for each site summarizing all statistical test results, which is used to rank sites from the most atypical to the least atypical [6]. The approach is based on the following principles:

(a) data coming from the various sites participating in a clinical trial should be largely similar, within normal variability limits (e.g., in multi-regional clinical trials) [15];

(b) a battery of standard statistical tests are applied to the patient data, where each test compares the distribution of the data in one site (“observed value”) with all study sites (“expected value”) (e.g., compare the mean systolic blood pressure of patients in one site to the mean across all study sites) [4];

(c) tests that are relevant given the type of each variable (continuous, categorical, or date variable) are systematically applied to all patient-level data in a completely unsupervised manner, regardless of their clinical importance, meaning or potential impact on the outcome of the trial [6]. For each variable in a trial between 1 and 8 statistical tests are applied;

(d) mixed-effects models (including both fixed and random effects) are used to allow for the natural variations between the sites, as data coming from all sites should be comparable and statistically consistent [5, 7, 14]; and.

(e) an overall “Data Inconsistency Score” (DIS) is computed for each site across all statistical tests performed on each clinical variable collected in the trial (at least 500 different *P*-values are typically computed for each site) to provide a summary metric at the site level. It is computed as the mean, on a log scale, of the *P*-values of all statistical tests performed. For each site, a weighted geometric mean is calculated with down-weighting of highly correlated tests and a resampling procedure is used to assign a *P*-value to the weighted geometric mean as described by Trotta et al. [6]. The DIS is the −*log*_*10*_*[P-value]* transformation. A DIS of 1.3 or larger corresponds to an overall *P*-value less than 0.05 and as such it flags a site whose data significantly differ from the data of all study sites. Note that the DIS is not adjusted for multiplicity, so it may flag more than 5% of the sites even if none are truly atypical. This feature of the SDM tool is considered desirable to err on the side of conservatism (i.e., flagging of too many sites for further inspection).

### Selection of Data

**Fig. 1:**
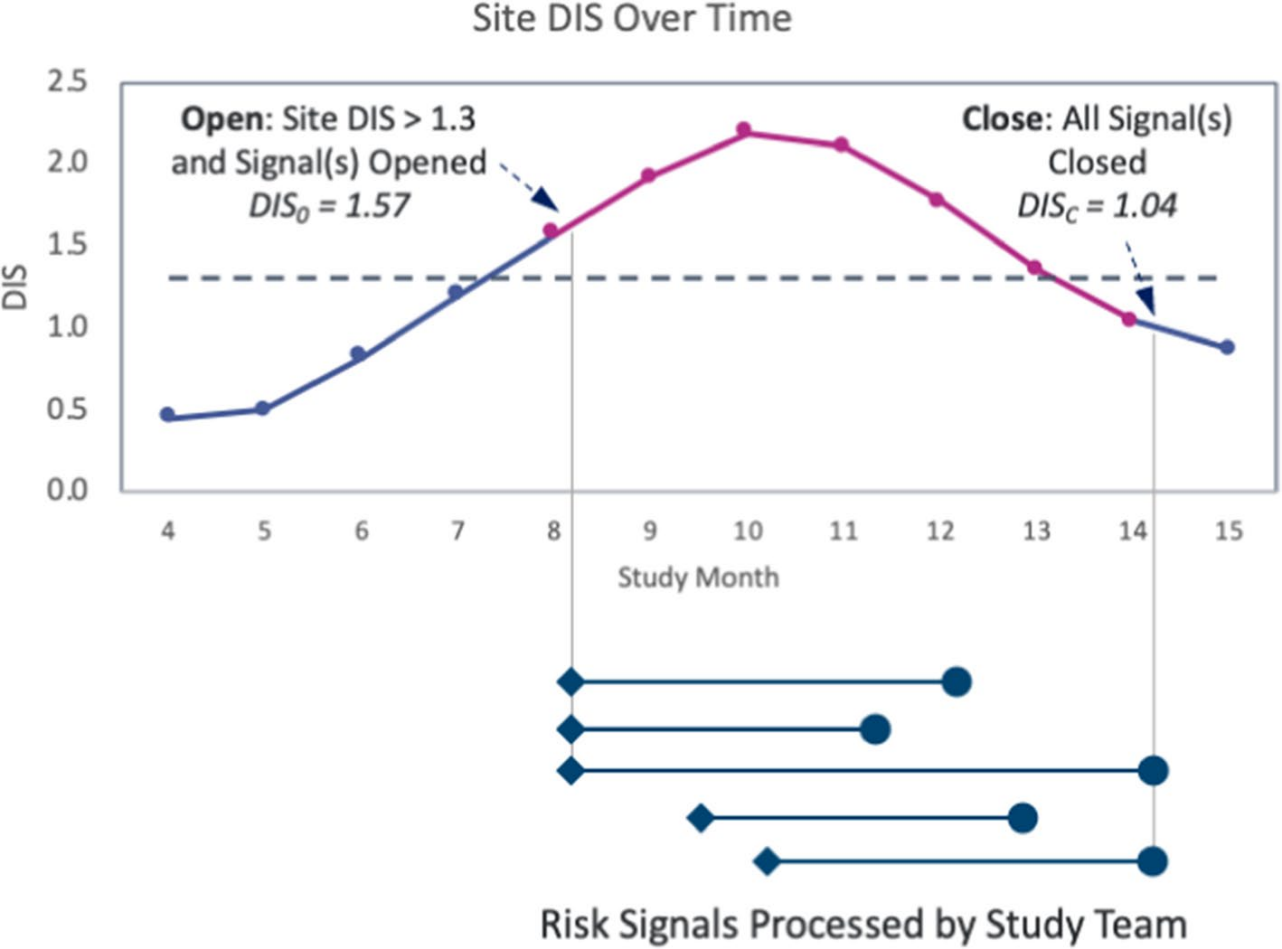
Illustration of site selection for the quality improvement analysis. Blue and Magenta DIS line: The line is magenta during the period of time that the study team is investigating and remediating issues at the site. The line is blue when no risk signals are being processed by the study team. Dotted Line: represents the 1.3 DIS threshold (representing a *P*-value of 0.05). Lines starting with a diamond and finishing with a circle: The diamond represents a signal being opened and the circle a signal being closed. *DIS* Data Inconsistency Score; *DIS*_*O*_ Opening DIS; *DIS*_*C*_ Closing DIS

**Fig. 2:**
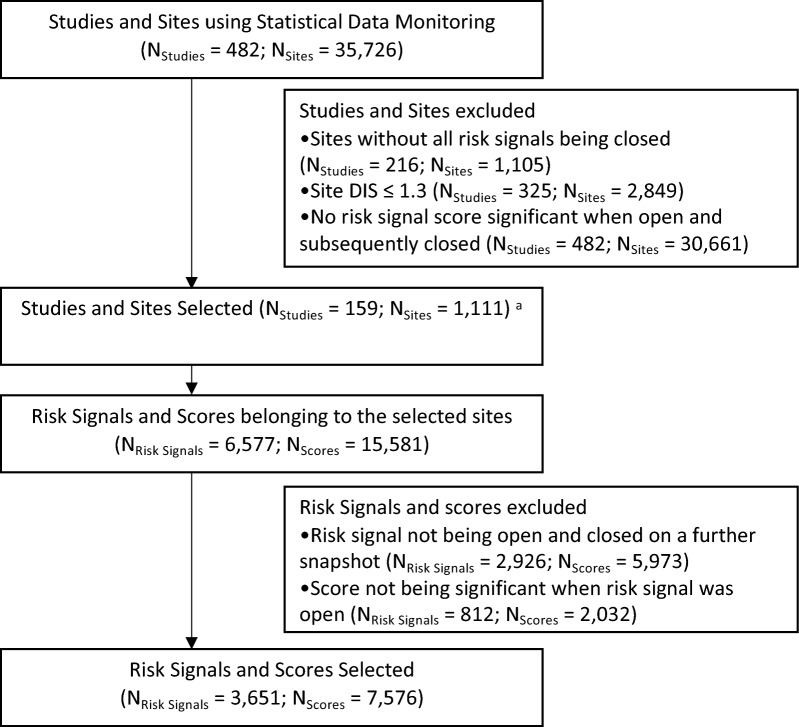
Study, Sites, Risk Signals and Scores Inclusion Flowchart. ^a^Some sites are selected in multiple analyses. There are 1111 distinct sites and 1264 distinct site analyses. *DIS* Data Inconsistency Score

The analysis was performed using data collected in the platform from September 1st, 2015 up to February 1st, 2023. The scope of the analysis included sites meeting the following criteria (Figs. 1 and 2):

1. Site belongs to a study that is completed and for which all risk signals were closed.
2. One or more risk signals were created for the site based on the SDM test results and were investigated (signal opened) and eventually closed by the study team.
3. The site’s DIS was > 1.3 at the time of risk signal(s) creation.

These criteria were defined to ensure availability of evidence covering the full history of each SDM risk signal processed by the study team from initiation through closure and where remediation of site issues and subsequent improvement of data quality would be an expected outcome.

### Quality Improvement Analysis

The first step in the analysis was to compute the following two quality improvement metrics for the selected sites:

Site DIS Improvement Rate: The total percent change in the site’s DIS from the time the site was first detected at risk (snapshot of data when the first risk signal was opened) until all risk signals at the site were closed by the study team (snapshot of data when the last risk signal is closed). The following formula was applied:

$$\frac{{ - \left( {DIS_{{\text{C}}} - DIS_{O} } \right)}}{{DIS_{O} }}$$

where: DIS_O_—Site’s DIS score when it exceeded 1.3 and at least one risk signal was open (“opening DIS”). DIS_C_—Site’s DIS score when all risk signals at the site were closed (“closing DIS”).

Figure 1 illustrates this formula with an example, where at month 8 a site was detected with a DIS over 1.3 (DIS_O_ = 1.57) and 3 signals were opened. Six months later, all risk signals for the site were closed (DIS_C_ = 1.04). The site DIS improvement rate in this example is $\frac{-(1.04 -1.57)}{1.57}$ which is equal to 34%. Note that in cases where a site’s DIS increases over this period of time (DIS_C_ > DIS_O_), the site DIS improvement rate will take on a negative value indicating a degradation in quality rather than an improvement.

Observed Value Improvement Rate: For each statistical test linked to a risk signal for a given selected site, the percent change of the observed value relative to the overall study estimate (i.e., expected value), from the time the risk signal was first opened until it was closed by the study team. The following formula is applied:

$$- \left[ {\frac{{\frac{{\left( {O_{C} { } - { }E_{C} } \right)}}{{E_{C} }}{ } - \frac{{\left( {O_{O} { } - { }E_{O} } \right)}}{{E_{O} }}{ }}}{{\frac{{\left( {O_{O} { } - { }E_{O} } \right)}}{{E_{O} }}}}} \right]$$

where: O_O_—The site’s observed value when the risk signal was opened. E_O_—The expected value when the risk signal was opened. O_C_—The site’s observed value when the risk signal was closed. E_C_—The expected value when the risk signal was closed.

**Fig. 3:**
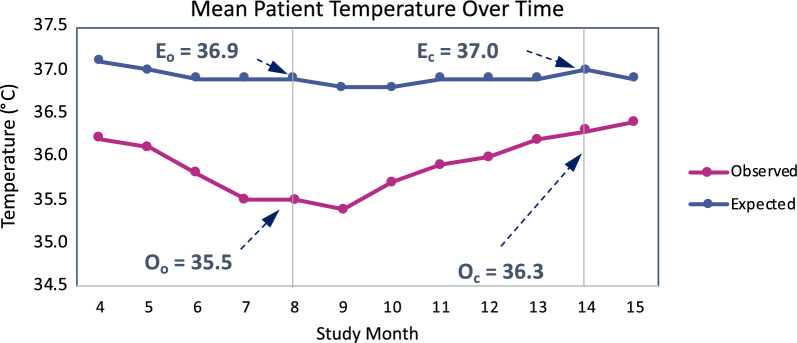
Illustration of Site Observed Value Improvement Rate Calculation. Magenta line represents the trend of the mean patient temperature in the site of interest (Observed value). The Blue line represents the expected value (overall study average) trend. The O_O_ represents the observed value of the site when the risk signal was created and the O_C_ the observed value of the site when the risk signal was closed. E_O_ and E_C_ represent the expected value (study overall average) when respectively the risk signal was opened and closed. *O*_*O*_ Opening Observed Value; *O*_*C*_ Closing Observed Value; *E*_*O*_ Opening Expected Value; *E*_*C*_ Closing Expected Value

Figure 3 illustrates the formula with an example of one observed value linked to a risk signal for a site. In particular, at month 8 the site was detected to have a low mean patient temperature (*O*_*O*_ = 35.5 °C) for their patients compared to the study average (*E*_*O*_ = 36.9 °C). Based on that finding, the study team opened a risk signal to investigate and remediate with the site as needed. The risk signal was closed six months later and the mean patient temperature at closure was significantly closer to the study average (*O*_*C*_ = 36.3 °C vs. *E*_*C*_ = 37.0 °C). The observed value improvement rate in this is example is

$$- \left[ {\frac{{\frac{{\left( {36.3 - 37.0} \right)}}{37.0} - \frac{{\left( {35.5 - 36.9} \right)}}{36.9}}}{{\frac{{\left( {35.5 - 36.9} \right)}}{36.9}}}} \right]$$

which is equal to 50%, meaning that the site’s observed value was 50% closer to the expected value than when the risk signal was first opened. Note that in cases where a site’s observed value moves further away from the study average over this period of time, the site observed value improvement rate will take on a negative value indicating a degradation in quality rather than an improvement.

Additionally, 95% Wilson score confidence intervals were used to estimate the rate of sites DIS improvement and observed value improvement [20].

### Comparison to Studies with No SDM

Data from two historical studies were available to us for which no central monitoring (i.e., SDM or KRIs) was performed during the conduct of the studies. These two studies were used for a comparison analysis to assess the difference in rate of site DIS improvement between studies using SDM and those not using SDM. Study 1 was a neurological study that included 60 sites and 7000 patients. Study 2 was a study in endocrinology that included 370 sites and 3500 patients.

A post-hoc SDM analysis was performed on the final completed study database for each of the two comparison studies, and then iteratively re-executed (retrospectively) on 3 versions of the trial database representing progressively earlier timepoints in the progression of each study. The calendar date by which a specified percentage of the total patient visits had been conducted was used as the cut-off date for each earlier version, and only patient data generated up to this calendar date were included in the analysis of that version.

**Fig. 4:**
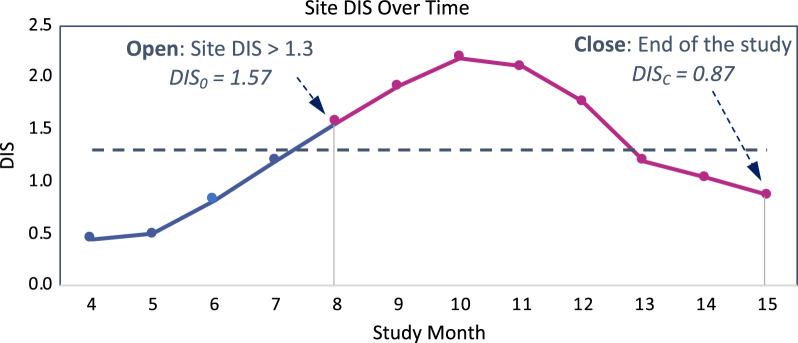
Illustration of site selection for the 2 studies with no SDM. Blue and Magenta DIS line: The line changes in magenta as from the first time the site has a DIS over 1.3 and up to the study completion. Line is Blue before the DIS becomes significant. Dotted Line: It represents the 1.3 DIS threshold (representing a *P*-value of 0.05). *DIS* Data Inconsistency Score; *DIS*_*O*_ Opening DIS; *DIS*_*C*_ Closing DIS

For each of the two studies, we calculated the Site DIS improvement rate using the same formula as in the main analysis, with slight adaptations of DIS_O_ and DIS_C_ definitions (Fig. 4):

- DIS_O_—Site’s DIS score when it first exceeded 1.3; i.e., the earliest database iteration at which this was observed. This represents the point in time when a study team would have typically opened risk signals for the site if central monitoring and SDM had been employed.
- DIS_C_—Site’s DIS score on the final completed study database. Since no risk signals were opened or closed for sites on these two studies, there is no meaningful milestone earlier than the end of the study at which to assess a site’s “closing” DIS. The DIS observed at the end of the study is used instead, which enables an assessment of the natural evolution of an “at-risk” site’s DIS in the absence of SDM.

Additionally, 95% Wilson score confidence intervals were used to estimate the DIS improvement in both studies [20].

## Results

In total, 1111 sites across 159 studies using SDM were selected (from 23 different sponsor and contract research organizations) (Table 1).

**Table 1:** Characteristics of the included studies

	Studies	Number of Patients	Number of Sites
	N (%)	Median [Interquartile range]	Median [Interquartile range]
Therapeutic Area			
Cardiovascular	7 (4.4)	869 [468–1,563]	87 [57–127]
Dermatology	22 (13.8)	391 [215–811]	88 [41–122]
Endocrinology	12 (7.6)	410 [195–783]	55 [35–76]
Gastroenterology	8 (5.0)	220 [142–633]	66 [31–121]
Infectious Disease	45 (28.3)	1,440 [630–2,428]	48 [25–93]
Musculoskeletal	6 (3.8)	191 [140–251]	59 [44–61]
Neurology	7 (4.4)	156 [130–191]	50 [31–55]
Oncology	31 (19.5)	279[135–610]	77 [28–135]
Respiratory	8 (5.0)	526 [310–628]	52 [34–133]
Other^a^	13 (8.2)	327 [210–509]	53 [39–63]
Study Phase			
Phase 1	13 (8.2)	744 [127–2,291]	29 [15–100]
Phase 2	37 (23.3)	238 [136–520]	39 [24–58]
Phase 3	98 (61.6)	611 [281–1,218]	75 [41–133]
Other^b^	11 (6.9)	281 [150–1,563]	31 [19–91]

^a^Group therapeutic areas in which less than 10 sites have been selected. It includes Mental Health and Addiction, Ophthalmology, Immunology, and Hematology

^b^Group study phases in which less than 10 sites have been selected. It includes Phase 4, Pre-Market Approval, and epidemiological studies

The overall landscape of clinical trials was fairly represented, with studies selected from a broad range of therapeutic areas and study sizes (number of patients and sites). Infectious disease was the highest represented therapeutic area with 45 studies (28%), which included a median of 1,440 patients and 48 sites. Additionally, all clinical phases were represented in the 159 studies selected from phase 1 (*N* = 13, 8.2%) to phase 3 (*N* = 98, 62%) (Table 1).

### Quality Improvement Analysis

Overall, a lower DIS (i.e., quality improvement) was observed in 83% of the sites (95% CI, 80–85%). Additionally, 64% of the sites had a closing DIS lower than 1.3. Across all sites, the site DIS improvement rate was 46% on average. Those results remained very similar across therapeutic areas and study phases (Table 2).

**Table 2:** Rate of sites with improved DIS and site DIS Improvement rate

	DIS Open	DIS Close	Sites with improved DIS		Sites with final DIS below 1.3	Site DIS Improvement rate (%)^a^
	Median [Interquartile range]	Median [Interquartile range]	N	% [95% CI]	N (%)	Median [Interquartile range]
**Main analysis—studies using SDM**						
Overall	1.74 [1.47–2.33]	1.00 [0.45–1.63]	1,044	82.6 [80.4, 84.6]	805 (63.7)	46.0 [11.5–75.3]
Therapeutic Area						
Respiratory	1.61 [1.44–2.01]	0.92 [0.45–1.96]	24	72.7 [55.8, 84.9]	21 (63.6)	52.8 [−2.2–77.7]
Infectious Disease	1.69 [1.47–2.23]	0.88 [0.39–1.50]	509	85.7 [82.6, 88.5]	403 (67.8)	50.4 [17.8–78.9]
Other^b^	1.65 [1.44–2.10]	0.87 [0.51–1.39]	76	84.4 [75.6, 90.5]	63 (70.0)	47.4 [12.3–68.8]
Dermatology	1.94 [1.58–2.78]	1.13 [0.45–1.92]	148	78.7 [72.3, 84.0]	105 (55.9)	47.2 [9.7–76.5]
Neurology	1.61 [1.41–1.85]	1.12 [0.46–1.46]	10	83.3 [55.2, 95.3]	8 (66.7)	46.7 [32.4–71.7]
Cardiovascular	1.59 [1.37–1.99]	0.95 [0.52–1.50]	13	81.2 [57.0, 93.4]	9 (56.3)	41.6 [0.7–71.9]
Gastroenterology	1.78 [1.49–2.49]	1.09 [0.58–1.67]	41	89.1 [77.0, 95.3]	28 (60.9)	43.8 [16.5–69.4]
Musculoskeletal	1.84 [1.48–2.23]	1.04 [0.54–1.62]	27	79.4 [63.2, 89.7]	21 (61.8)	42.2 [8.4–69.1]
Oncology	1.85 [1.50–2.44]	1.14 [0.61–1.70]	106	80.9 [73.3, 86.7]	81 (61.8)	37.3 [7.2–72.6]
Endocrinology	1.77 [1.47–2.23]	1.18 [0.68–2.00]	90	75.0 [66.6, 81.9]	66 (55.0)	33.9 [0.5–61.5]
Study Phase						
Phase 3	1.78 [1.48–2.45]	0.93 [0.42–1.67]	691	82.8 [80.0, 85.2]	530 (63.5)	49.4 [11.8–78.0]
Phase 1	1.67 [1.46–2.12]	1.01 [0.47–1.62]	134	85.9 [79.6, 90.5]	101 (64.7)	43.7 [12.5–72.6]
Other^c^	1.69 [1.52–1.92]	0.93 [0.46–1.17]	19	86.4 [66.7, 95.3]	17 (77.3)	42.2 [31.4–76.1]
Phase 2	1.66 [1.45–2.02]	1.07 [0.54–1.56]	200	79.7 [74.3, 84.2]	157 (62.5)	40.7 [9.1–69.6]
**Comparison—studies not using SDM**						
Overall	1.77 [1.45–2.45]	1.46 [0.30–3.75]	24	55.8 [41.1, 69.6]	21 (48.8)	16.7 [−40.7–77.7]
Study 1	1.46 [1.38–1.66]	1.48 [1.20–2.20]	3	37.5 [13.7, 69.4]	2 (25.0)	50.6 [−43.8–82.0]
Study 2	1.96 [1.49–2.81]	0.91 [0.25–3.85]	21	60.0 [43.6, 74.4]	19 (54.3)	2.7 [−7.5–12.0]

^a^Negative value indicating a degradation in quality rather than an improvement

^b^Group of therapeutic areas in which less than 10 sites have been selected. It includes Mental Health and Addiction, Ophthalmology, Immunology, and Hematology

^c^Group of study phases in which less than 10 sites have been selected. It includes Phase 4, Pre-Market Approval, and epidemiological studies

*DIS* Data Inconsistency Score, *95% CI* 95% Wilson Score Confidence interval

For the two comparison studies (for which central monitoring including SDM had never been used), a lower DIS was observed in 56% of the sites (95% CI, 41–70%) and the site DIS improvement rate was 17% (Table 2).

For the sites with improving DIS, 71% of the observed values moved closer to the expected values and 51% of them were no longer statistically significant when the risk signal was closed. Note that 20% of the observed values had no change in the number of records from risk signal open to close. Hence, in these cases there was no opportunity for the observed values to improve except for the possibility of data entry corrections to existing data records. Additionally, the observed values were on average 45% closer to the expected values when the risk signal was closed. The rate of improving observed values remained very similar across the different statistical tests and dataset domains (Table 3).

**Table 3:** Rate of observed values that improved and observed value improvement rate among sites with an improved DIS

	Obs. values where N did not change	Observed values that improved^a^		Observed values that improved below 1.3	Observed Value Improvement Rate (%)^a^
	N (%)	N	% [95% CI]	N (%)	Median [Interquartile range]
Overall	1,137 (20.0)	4,036	71.1 [69.9, 72.3]	2,884 (50.8)	44.5 [4.7–94.6]
Dataset Domain					
Disposition	43 (26.7)	122	75.8 [68.6, 81.7]	106 (65.8)	85.2 [32.5–﻿100.6]
Immunogenicity Specimen Assessments	12 (17.9)	56	83.6 [72.9, 90.6]	41 (61.2)	73.5 [42.5–100.0]
Procedures	23 (18.5)	105	84.7 [77.3, 90.0]	89 (71.8)	66.8 [30.4–101.1]
Exposure	51 (17.6)	209	72.3 [66.9, 77.2]	167 (57.8)	66.6 [11.9–101.2]
Demographics	25 (26.9)	59	63.4 [53.3, 72.5]	45 (48.4)	66.5 [0.0–101.2]
Subject Visits	6 (6.0)	67	67.0 [57.3, 75.4]	53 (53.0)	66.5 [8.4–96.7]
Healthcare Encounters	19 (32.2)	37	62.7 [50.0, 73.9]	32 (54.2)	59.9 [7.3–94.1]
Physical Examination	22 (9.5)	189	81.5 [76.0, 85.9]	124 (53.4)	59.0 [11.7–101.3]
Concomitant Medications	68 (19.0)	249	69.7 [64.8, 74.3]	184 (51.5)	54.1 [1.9–101.1]
Adverse Events	64 (17.8)	262	72.6 [67.8, 76.9]	206 (57.1)	56.4 [11.6–101.7]
Concomitant Medications	68 (19.0)	249	69.7 [64.8, 74.3]	184 (51.5)	54.1 [1.9–101.1]
Other^b^	86 (18.7)	317	68.9 [64.5, 73.0]	235 (51.1)	51.4 [2.6–96.0]
Medical History	81 (32.3)	171	68.1 [62.1, 73.6]	135 (53.8)	48.3 [0.2–100.3]
Clinical Events	52 (22.0)	160	67.8 [61.6, 73.4]	119 (50.4)	48.1 [3.3–88.0]
Laboratory Test Results	132 (19.7)	459	68.4 [64.8, 71.8]	335 (49.9)	45.7 [3.1–96.9]
ECG Test Results	65 (35.5)	132	72.1 [65.2, 78.1]	99 (54.1)	41.8 [9.0–91.9]
Vital Signs	159 (19.3)	600	72.8 [69.7, 75.7]	393 (47.7)	34.8 [2.9–76.2]
Inclusion/Exclusion Criteria	22 (43.1)	28	54.9 [41.4, 67.7]	21 (41.2)	34.4 [0.4–98.8]
Questionaires	136 (18.5)	528	71.6 [68.3, 74.8]	339 (46.0)	30.9 [3.3–74.2]
Disease Response	71 (16.9)	286	68.3 [64.4, 73.2]	161 (38.4)	21.0 [4.0–47.7]
Statistical Test					
Count	36 (9.1)	345	87.1 [83.5, 90.1]	307 (77.5)	94.9 [50.7–101.1]
Data Reporting^c^	413 (18.8)	1,631	74.3 [72.4, 76.1]	1,281 (58.4)	68.0 [12.6–101.3]
Categorical	306 (21.3)	904	62.9 [60.4, 65.4]	605 (42.1)	33.4 [0.8–77.4]
Continuous	382 (23.2)	1,156	70.2 [67.9, 72.3]	691 (42.0)	23.7 [1.6–60.1]

^a^Improvement measured by comparing the gap between observed and expected at open and at close. Only gap reduction is considered as an improvement

^b^Group dataset domains in which less than 50 observed values have been selected

^c^Data reporting tests include tests for missing data and reporting rates by patients and visits (count of records per patients or patient-visits)

*95% CI* 95% Wilson Score Confidence interval

### Two Sample Sites

**Fig. 5:**
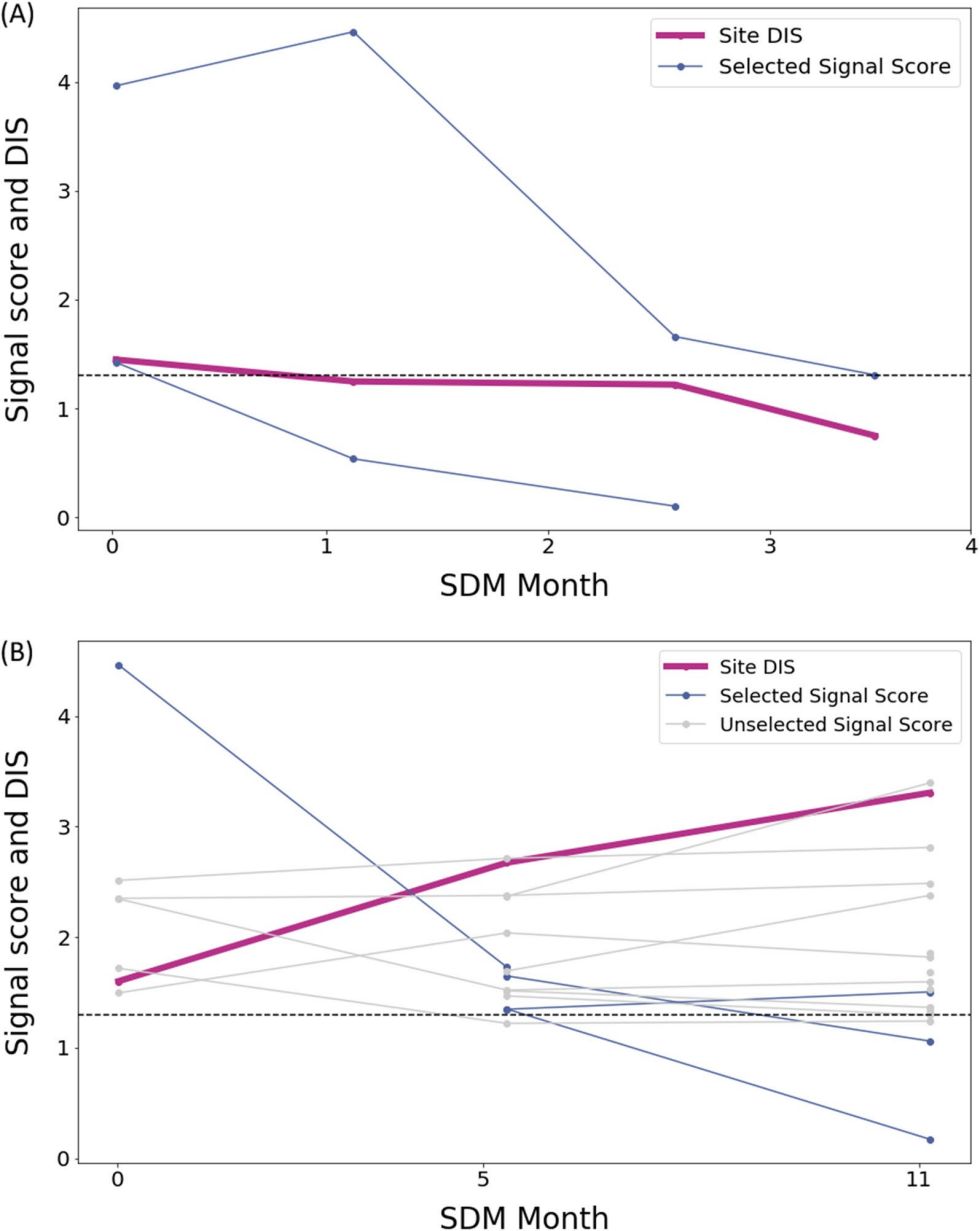
**A** Improving and **B** Non-improving Site Examples. Circles on the selected signals score lines represent the analysis of a new increment of study data. The same increment were used for the DIS calculation

Figure 5 displays the evolution of the DIS and risk signal scores for two sample sites from this analysis. The first site shows a DIS improvement and the second one a worsening DIS. Figure 5.A shows a site with improving DIS and risk signal scores in a dermatology study. The site was first flagged with a DIS of 1.45 (*P* = 0.035). Two risk signals were created and both risk signals were no longer significant at the time of risk signal closure. Additionally, when the risk signals were closed, the DIS was no longer statistically significant (DIS_C_ = 0.75, *P* = 0.18). The first risk signal represented a very high disease response rate. After investigation, it appears that it was due to a data entry error. The error was corrected and at the time of the risk signal closure 39 additional disease response scores were added. The second risk signal flagged a low volume of drug dispensation among the different patients of the site. The Clinical Research Associate (CRA) checked weighing techniques and the calibration of the tool. After investigation, the issue was due to a misunderstanding of the reporting requirements. At risk signal closure, no additional erroneous results were reported.

Figure 5.B shows a site in a gastroenterology study with a DIS that did not improve (DIS_O_ = 1.6, *P* = 0.025; DIS_C_ = 3.31, *P* = 0.0005). In that site, a total of 19 risk signals were created. Fifteen risk signals were not selected in the current analysis as they were all immediately closed (i.e., not investigated as they do not represent quality risk for the study team). Those risk signals clearly described a site with an atypically unhealthy population. Additionally, the site belonged to a country with specific protocol requirements in which some assessments were not applicable. Neither of these explanations represented data quality issues and therefore, as expected, most of the 15 risk signals did not show improvement at closure. The remaining 4 risk signals were investigated by the study team (i.e., opened) and 3 of them improved at closure. Those risk signals flagged missing data along with AEs that were not reported when expected. At the time of risk signal closure, the AE reporting rate increased and the missing data were provided.

## Discussion

Central monitoring, including the use of SDM software, is generally designed for the purpose of continually identifying sites that are deviating from an expected pattern of quality behavior, so that study teams can intervene at those sites and address any confirmed issues [1, 2, 10]. The results of the current analysis provide clear evidence that a majority of the sites flagged by this approach show a significant level of quality improvement, across all therapeutic areas and study phases.

This conclusion relies on the premise that the two metrics used in this analysis are valid indicators of quality improvement. Site DIS provides a measure of the overall level of atypicality of the patient data (i.e., risk data quality issue) reported from each site, which is not by itself a conclusive indicator of poor quality. Indeed, as shown in the second example (Fig. 5.B), some sites will have a high DIS because they enrolled an atypical group of patients (e.g., older and more severe condition or disease at baseline) which the study team determines does not represent an actual quality issue. Nevertheless, when investigation of atypical data patterns leads to the confirmation of quality issues at a site, those atypical data patterns become a definitive indicator of poor quality. It is then clearly expected that remediation of the identified issues should result in generation of less atypical data at the site and a correspondingly lower site DIS. The same expectation follows for the site’s observed values on which the data atypicality is measured; i.e., those values should move closer to the expected estimate across all sites in the study. For example, if the rate of patient adverse events (AEs) reported at a site was atypically low and confirmed to be an issue (e.g., site mistakenly thought that they were only supposed to report serious AEs), re-training of the site should result in a subsequent increase in the observed AE reporting rate bringing it closer to the average rate across the study.

A theoretical concern exists that sites observed with a high DIS at one timepoint will naturally tend toward a lower DIS subsequently due to a regression-to-the-mean effect [21]. Indeed, selecting sites with a high DIS means by definition that we are selecting sites at the tail of the distribution. Therefore, by play of chance, there is a high probability that the DIS of the same site becomes less extreme in subsequent timepoints, resulting in an improving DIS for the site Without taking into account the regression-to-the-mean effect, the baseline assumption (i.e., if central monitoring had not been used) is that a site DIS has a 50% chance on average to improve, as a coin flip probability. The results of the analysis on two historical studies not using SDM showed that only slightly more than half of the sites (56%, 95% CI 41–70%) with an initially high DIS were observed to have a lower DIS at study closure, which points to a dominant effect of regression-to-the-mean. However in studies using SDM, this rate increased to 83% (95% CI 80–85%). The confidence intervals from studies not using SDM and those using SDM do not overlap, which suggests that DIS improvements are seen as a result of the SDM approach. However, the non-randomized nature of this comparison, and the limited evidence from studies not using SDM, both call for caution in interpreting this observed difference.

While the results of the current analysis are quite positive—83% of sites with improved DIS and 71% of observed values improving—one might ask why the level of improvement was not even better than this? In particular, 17% of the flagged sites did not end up with an improved DIS and 29% of the observed values did not improve. There are actually multiple factors that explain why some sites do not show improvement. First, as previously mentioned, data atypicality is not a definitive indicator of poor quality and in some cases, the observed atypicality is found to be explainable and simply does not reflect a quality issue. In these cases we would not expect the observed atypicalities to moderate on average following study team review. This is illustrated in a study in which a site recruited mostly older (though still eligible) patients who accordingly had a higher number of medical histories and higher rate of safety and efficacy findings [3].

A second reason for lack of observed improvement is in situations where, by the time the data atypicalities at a site are investigated and issues confirmed, all patients at that site have completed their participation in the study. In such cases there is no further patient data to be generated and/or reported from the site and therefore no opportunity to observe improvement in the data. While we could not quantify the contribution of these two factors on the overall results of our study, it can be hypothesized that they explain some of the observed non-improvement and that the actual rate of improvement is higher than that observed.

We observed no marked difference in the rate of improving sites or the size of improvement across the different therapeutic areas or study phases. This supports a conclusion that SDM is beneficial in a broad range of clinical trials, which is consistent with FDA and EMA recommendations [1, 2]. All data collected during a clinical trial are at risk of data quality issues [4, 14, 15]. However, some factors may increase those risks, including the following: complex study protocols, complicated eCRF and database designs, and poor site training [22, 23]. This is why identifying and controlling risks related to a clinical trial both prior to and during the trial is essential.

A limitation of the current analysis is that we assessed metrics of improved quality only for risks that were identified by the SDM solution and subsequently acted upon by the study team. Therefore, the analysis did not assess how effective SDM is at identifying all of the relevant issues in a clinical trial. Instead, it assesses to what extent study team follow-up on identified risks is resulting in improved quality.

A second limitation is related to the comparison with studies that did not use SDM, for which data from only two studies were available. Although the comparison is based on a more limited volume of data, the results do suggest that the significant level of improvement observed for SDM studies is not due to the play of chance.

This paper complements a previous paper showing that the use of KRIs was effective at improving quality in clinical trials. As mentioned in that paper, "it is important to recognize that improved quality does not come automatically through implementation of central monitoring. The degree of success achieved is highly dependent on the thoughtful design and implementation of all central monitoring tools (including KRIs) and risk follow-up processes.” [16].

## Conclusion

These results provide quantitative evidence that central monitoring including SDM, which is recommended by regulatory agencies [1, 2], is resulting in improved quality. When properly implemented, managed and followed-up, SDM enables a targeted approach to identifying and addressing emerging quality-related risks during a study.

## Conflict of interest

SdV, LT, WS, and SY are employees of CluePoints. LT, SY, and MB hold stock in CluePoints.

## Data Availability

Not applicable.
